# The Molecular Mechanisms of Iron Metabolism and Its Role in Cardiac Dysfunction and Cardioprotection

**DOI:** 10.3390/ijms21217889

**Published:** 2020-10-24

**Authors:** Tanya Ravingerová, Lucia Kindernay, Monika Barteková, Miroslav Ferko, Adriana Adameová, Vladislava Zohdi, Iveta Bernátová, Kristina Ferenczyová, Antigone Lazou

**Affiliations:** 1 Institute for Heart Research, Centre of Experimental Medicine, Slovak Academy of Sciences, 9 Dúbravská Cesta, 84104 Bratislava, Slovak Republic; lucia.griecsova@gmail.com (L.K.); monika.bartekova@savba.sk (M.B.); miroslav.ferko@savba.sk (M.F.); aadameova@gmail.com (A.A.); kristina.ferenczyova@gmail.com (K.F.); 2 Department of Pharmacology and Toxicology, Faculty of Pharmacy, Comenius University in Bratislava, 10 Odbojárov st., 83232 Bratislava, Slovak Republic; 3 Institute of Anatomy, Faculty of Medicine, Comenius University in Bratislava, 2 Sasinkova st., 81108 Bratislava, Slovak Republic; vzohdi@gmail.com; 4 Institute of Normal and Pathological Physiology, Centre of Experimental Medicine, Slovak Academy of Sciences, 1 Sienkiewiczova st., 81371 Bratislava, Slovak Republic; iveta.bernatova@savba.sk; 5 Laboratory of Animal Physiology, School of Biology, Aristotle University of Thessaloniki, 54124 Thessaloniki, Greece

**Keywords:** iron, myocardial ischemia/reperfusion injury, reactive oxygen species, mitochondria, ferroptosis, cardioprotection

## Abstract

Iron is an essential mineral participating in different functions of the organism under physiological conditions. Numerous biological processes, such as oxygen and lipid metabolism, protein production, cellular respiration, and DNA synthesis, require the presence of iron, and mitochondria play an important role in the processes of iron metabolism. In addition to its physiological role, iron may be also involved in the adaptive processes of myocardial “conditioning”. On the other hand, disorders of iron metabolism are involved in the pathological mechanisms of the most common human diseases and include a wide range of them, such as type 2 diabetes, obesity, and non-alcoholic fatty liver disease, and accelerate the development of atherosclerosis. Furthermore, iron also exerts potentially deleterious effects that may be manifested under conditions of ischemia/reperfusion (I/R) injury, myocardial infarction, heart failure, coronary artery angioplasty, or heart transplantation, due to its involvement in reactive oxygen species (ROS) production. Moreover, iron has been recently described to participate in the mechanisms of iron-dependent cell death defined as “ferroptosis”. Ferroptosis is a form of regulated cell death that is distinct from apoptosis, necroptosis, and other types of cell death. Ferroptosis has been shown to be associated with I/R injury and several other cardiac diseases as a significant form of cell death in cardiomyocytes. In this review, we will discuss the role of iron in cardiovascular diseases, especially in myocardial I/R injury, and protective mechanisms stimulated by different forms of “conditioning” with a special emphasis on the novel targets for cardioprotection.

## 1. Introduction

Iron is an essential element in all living organisms. Iron is intimately involved in a wide range of biological processes, including oxygen transport via hemoglobin in the red blood cells, DNA synthesis, cellular respiration, and electron transfer, as well as overall metabolism [1,2].

The human body contains about 2–5 g of total iron, the majority of which is intracellular. Of that amount of iron, approximately 60–80% is bound to heme in hemoglobin and myoglobin and to various nonheme enzymes and proteins. Further, 20–40% of iron is bound to specialized iron-storage proteins such as ferritin (FT) or hemosiderin. The extracellular iron constitutes only about 0.1% of the total body iron and is mainly bound to the iron transport protein in the serum named transferrin (Tf).

In humans, dysregulation of iron metabolism can result in both low and high iron levels. Iron deficiency is frequently present in heart failure patients with reduced ejection fraction associated with low exercise capacity and reduced quality of life. Oral iron products have been shown to be non-efficient in these patients with the option of intravenous administration. The current knowledge of the pathophysiology of iron deficiency in heart failure, its clinical impact, and possible treatment options are extensively discussed in the review paper by von Haehling et al. (2019) [3]. In line, it has been shown that iron deficiency impairs contractility of human cardiomyocytes through decreased mitochondrial function and lower energy production, leading to impairment of heart function [4]. In addition, systemic iron deficiency is associated with a higher incidence of coronary artery disease and myocardial infarction (MI) in the population [5], as well as with a higher mortality rate in these patients [6].

Low iron levels also lead to absolute iron deficiency and/or anemia while chronic inflammation or cancer can result in functional iron deficiency and anemia of chronic diseases [7,8,9,10].

On the other hand, iron overload resulting from genetic disorders or from oral or parenteral iron treatment are also considerable health problems due to the risk of serious organ damage, including cancer [11,12]. Cardiomyopathy is the second leading cause of mortality in patients with hereditary hemochromatosis [12]. In addition, disorders of iron metabolism participate also in the mechanisms of other diseases, such as type 2 diabetes, obesity, and non-alcoholic fatty liver disease [13]. Regarding the cardiovascular system, iron overload can cause impaired vascular function and aggravate the development of atherosclerosis, arrhythmias, and heart failure, as well as overall morbidity of patients [6,12,14].

Concentrations and homeostasis of iron in the organism are regulated by several mechanisms responsible for the control of intracellular iron metabolism, transfer, uptake, and export, as well as intracellular storage (extensively reviewed in [15,16]). Uncontrolled increase of iron concentration (of genetic and nongenetic “transfusional” origin) is associated with a failure of these mechanisms to maintain a balance in iron concentration resulting in iron overload that represents a potential danger to the function of basic cellular mechanisms [17,18]. Redox properties of iron facilitate the production of reactive oxygen species (ROS) including the most toxic hydroxyl radical [19,20]. Moreover, both ferrous (Fe^2+^) and ferric (Fe^3+^) iron mediate lipid peroxidation, resulting in the formation of alkoxyl (RO) and peroxyl (RO_2_) radicals [21].

It has been also demonstrated that increased mitochondrial iron-related reactive oxygen species (ROS) production contributes to myocardial injury in animal models of ischemia/reperfusion (I/R) [22] and in human cardiac tissue samples obtained from patients with ischemic cardiomyopathy [23]. Although it was hypothesized already in 1981 that iron may represent a further risk factor of cardiovascular diseases [24], this issue is still not completely resolved.

## 2. Systemic Iron Homeostasis

The iron of the body is constantly recycled, mainly through normal phagocytosis of aged erythrocytes by macrophages. The iron contained in the hemoglobin of these erythrocytes is released back to the plasma to be reuptaken either by bone marrow for the synthesis of new erythrocytes or by other organs to synthesize iron-containing molecules. Under physiological conditions, in the absence of blood loss, only small amounts of iron (1–2 mg/day) are lost due to desquamation of epithelial cells, and these losses are replenished by the uptake of dietary iron.

Dietary iron is absorbed by epithelial cells of the small intestine by two distinct mechanisms: The heme form of iron is absorbed at the apical site of the epithelial cell via a specific heme transporter called heme carrier protein 1 (HCP1) [25] while the nonheme insoluble Fe^3+^ is first reduced to Fe^2+^ by cytochrome b reductase 1 (DCYTB) and then transported by divalent metal transporter 1 protein (DMT-1) across the membrane [26]. Once transported into the cell, heme-iron is liberated by hemoxygenase-1 (HO-1), which degrades heme to Fe^2+^, biliverdin, and carbon monoxide (CO). Ferrous iron can be stored in the ferritin cores or released to circulation via ferroportin (FPN) at the basolateral membrane of enterocyte [15]. Up to now, FPN is the only known iron transporter that causes iron efflux from various cells (enterocytes, macrophages, or peripheral cells) [27,28]. Further distribution of iron to peripheral tissues is mediated by Tf, which can transport one or two ferric ions. Tf binds to Tf receptors (TfR) on the cell membrane followed by internalization of the Tf-TfR complex and iron release into the cell [15]. In physiological conditions, Tf saturation by iron is about 30% and, thus, Tf serves as a buffer of potentially toxic nontransferrin-bound iron (NTBI).

When Tf saturation is increased, elevated levels of NTBI corresponding mainly to circulating potentially toxic iron species become a leading cause of damage both at the cellular and the intracellular level, due to iron overload and its high propensity to trigger ROS production [29,30]. Iron overload, either at the systemic or at the cellular levels, is also associated with harmful effects on cardiac function as has been demonstrated in animal studies using mice with cardiomyocyte-specific deletion of the FPN gene as well as in humans with hemochromatosis or β-thalassemia [30,31]. Furthermore, patients with Freidreich ataxia, a disease characterized by mitochondrial iron overload in the heart and brain, develop dilated cardiomyopathy and arrhythmias [32].

Iron homeostasis in the body is tightly regulated. Main iron flows among the digestive tract (duodenum), plasma, erythrocytes, macrophages, liver (hepatocytes), and spleen and other cell types are regulated by hormone hepcidin encoded by the hepcidin antimicrobial peptide (HAMP) gene, which is the main regulator of iron homeostasis. Hepcidin, a 25-amino acid protein, is released mainly in the hepatocytes, but its release was detected also in other tissues, such as the heart. The role of hepatic hepcidin is well known. It regulates FPN-mediated iron efflux by promotion of FPN internalization and degradation in conditions of high levels of iron. Hepcidin release is increased by iron overload, red blood cell transfusion, by iron treatment, and genetic factors as well as by infection and/or inflammation. On the other hand, hepcidin release is decreased mainly by hypoxia, elevated erythropoiesis, or iron deficiency but also by sex hormones [33,34,35]. At the molecular level, hepcidin release is negatively regulated via bone morphogenetic protein/small mothers against decapentaplegic (BMP-SMAD) pathway, activated in a paracrine manner by BMP2 and BMP6, produced by liver sinusoidal endothelial cells, and liver sinusoidal endothelial cells were suggested as iron sensors [36,37]. Moreover, it was recently suggested that hepcidin serum levels may be increased by leptin, a hormone which is dysregulated in metabolic disorders [38].

In addition to regulation by intracellular iron level, iron metabolism is also regulated by several iron-independent mechanisms. Oxidative stress increases nuclear factor erythroid 2-related factor 2 (Nrf2) synthesis to activate Nrf2-mediated antioxidant defense system [39]. Concurrently, several genes involved in iron metabolism are regulated by Nrf2: Ferritin heavy chain (FTH), ferritin light chain (FTL), Tf, FPN, and HO-1 [40]. Nrf2-mediated mechanism regulates also hepcidin synthesis via modulation of BMP6 [41]. Another factor affecting iron metabolism is inflammation associated with IL-6-mediated increase in hepatic hepcidin synthesis resulting in the degradation of FPN [42].

## 3. Cellular Iron Homeostasis in the Heart

### 3.1. Uptake and Cellular Regulation of Iron

Iron can enter cardiomyocytes in several ways. The uptake of Tf-bound iron is mediated through binding to TfR1 and subsequent internalization by endocytosis. The acidic environment of the lysosome liberates iron from the Tf-TfR1 complex and iron is transported into the cytosol, whereas the Tf-TfR1 complex is recycled to the cell surface [34]. Another important pathway for the influx of iron into the cells is through DMT-1 protein, which mediates the import of non-Tf-bound iron. In addition, non-Tf-mediated iron uptake in cardiomyocytes can also occur through the L-type and the T-type calcium channels present in cardiac plasma membrane and through zinc transporters [43,44].

Upon entry into the cell, iron becomes a part of the poorly characterized labile iron pool (LIP) in the cytosol. Iron in the LIP acts as an intermediate and can be utilized for storage in FT (where it is redox inert) or go through biosynthetic pathways to generate heme and iron-sulfur clusters in the mitochondrion or iron-requiring proteins in the cytosol [26]. The excess iron is removed from the cell by the Fe^2+^ exporter, ferroportin 1 (FPN1) [16,31]. Under normal conditions, the level of labile iron is kept very low to prevent ROS formation. However, pathological states of iron overload can dramatically increase the labile iron pool.

Intracellular FT is the ubiquitous protein that serves as a type of buffer that protects against iron deficiency and excess. FT can store as much as 4500 atoms of iron (mode value of ∼1500 atoms) in a soluble and nontoxic form and can transport it to areas where it is required [45]. FT is important for iron metabolism since it binds and sequesters iron in conditions of iron overload and releases it in the case of iron deficiency. Under iron deficiency conditions, FT is degraded and releases stored iron, which can be used for the most crucial processes, whereas in iron overload conditions it stores iron in a safe form to prevent the induction of oxidative stress. Iron sequestering property of FT is considered as a source of antioxidant effects [46]. Human FT structure consists of 24 subunits of two proteins chains: FTH protein and FTL protein. FTH-to-FTL ratio varies in various cell types and depends on tissue state [47]. FTH participates in the maintenance of LIP and protects the cell against oxidative stress due to high levels of Fe^2+^ available for the Fenton reaction. On the other hand, FTL, in the form of apoferritin, is present also in blood; serum FTL concentrations correlate with various pathological states [48].

Cellular iron homeostasis is regulated by a post-transcriptional mechanism through interaction of iron regulatory proteins (IRP) 1 and 2 with iron-responsive elements (IRE) on mRNA of the respective genes, which modulate the synthesis of key iron metabolism proteins involved in iron uptake, storage, and release [49]. While IRP1 possess dual function, as IRE-binding protein and cytosolic aconitase (c-aconitase, CA), IRP2 is stable in hypoxic and iron-deficient cells but degraded by the proteasome in normoxic cells [50]. Under conditions of low cellular iron concentration, IRPs stabilize the mRNA of TfR1 and DMT-1 to promote iron influx; at the same time, they inhibit mRNA translation of FPN1 and FT to inhibit iron efflux and storage, respectively [51,52].

In cardiac myocytes, FPN1 is also regulated by hepcidin, both by that produced by the liver and produced locally in the heart. Hepcidin causes degradation of FPN1 resulting in a smaller iron efflux from the cardiomyocyte. Cardiac hepcidin has important autocrine effects and participates in the autonomous regulation of iron in cardiomyocytes that are distinct from systemic iron regulation. In contrast to systemic hepcidin, cardiac-hepcidin protein is upregulated rather than downregulated by hypoxia to preserve cellular iron [53]. This mechanism involves post-transcriptional regulation of hepcidin peptide by the hypoxia-inducible factor (HIF) system through upregulation of furin, a HIF1α target gene [54]. The fact that cardiomyocytes are equipped with redundant iron-importing mechanisms, while only one iron-exporting protein, FPN, is available, explains the high susceptibility of the heart to iron overload. Iron homeostasis is under substantial subcellular regulation within cardiomyocytes, whereas iron utilization and transport in the mitochondrial compartment, which represents the metabolic engine of the heart, is controlled by a distinct set of proteins, such as mitochondria-specific ferritin (FTMT), frataxin, and mitochondrial ATP-binding cassette subfamily B (ABCB) transporters, as discussed later [55,56].

**Figure 1:**
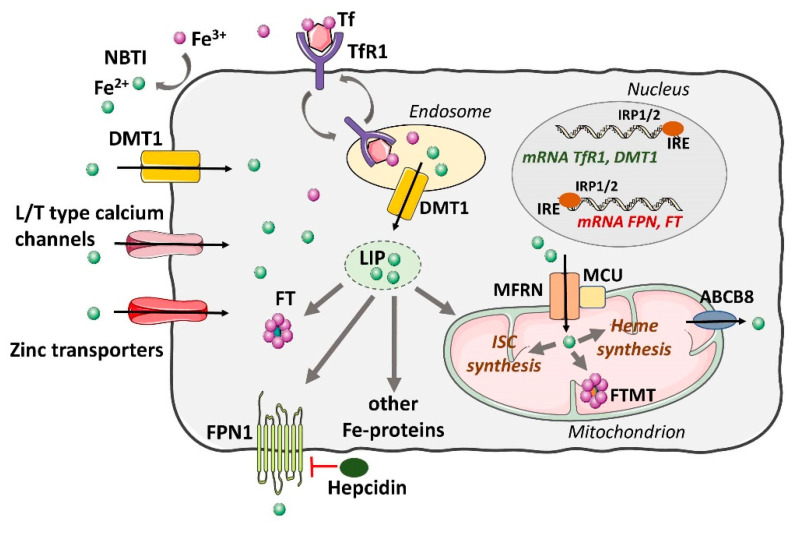
Schematic representation of iron homeostasis in cardiac myocytes. Tf-bound iron is imported into the cell through binding to transferrin receptor 1 (TfR1) and subsequent internalization by endocytosis. Upon liberation of iron, the Tf-TfR1 complex is recycled to the cell membrane. Non-Tf-mediated iron uptake occurs through L/T-type calcium channels, zinc transporters, and DMT-1. In the cytosol, iron is bound by the storage molecule FT and a small amount remains as labile iron. Iron is exported from the cell via FPN, which is regulated by the hormone hepcidin. Iron is transported into the mitochondria through MFRN and MCU and can be utilized for the synthesis of heme and iron-sulfur clusters or for storage FTMT. The ABCB8 transporter serves as an iron exporter. IRP1/2 proteins control iron homeostasis in cardiac myocytes. IRPs bind to IRE sites in the mRNA of DMT-1 and TfR1, leading to their stabilization, and of FT and FPN, leading to their inhibition. Abbreviations: ABCB8, adenosine triphosphate (ATP)-binding cassette subfamily B member 8; DMT-1, divalent metal transporter 1; FPN, ferroportin; FT, ferritin; FTMT, mitochondrial ferritin; IRE, iron-responsive element IRP, iron regulatory protein; ISC, iron-sulphur cluster; LIP, labile iron pool; MFRN, mitoferrin; MCU, mitochondrial calcium uniporter; NTBI, nontransferrin-bound iron; Tf, transferrin; TfR1, transferrin receptor 1.

Mechanisms of Tf-dependent and non-Tf-dependent mechanisms of iron import into the cardiomyocytes, iron storage, and export from the cells, as well its transport to the mitochondria and further removal, are schematically represented in Figure 1.

### 3.2. The Role of Iron in Heart Mitochondria and Cardiomyocyte Dysfunction

Mitochondria are known for their key role in energy production. However, it is less recognized that they also play an important role in iron metabolism [55,56,57] and in the modulation of cardiac damage during ischemia and reperfusion [58]. In fact, the mitochondrion is the sole site of heme synthesis and a major generator of iron sulfur clusters (ISCs), both of which are present in mitochondria and cytosol. Heme- and ISC-containing proteins are integral parts of Mitochondrial Oxidative Phosphorylation System (OXPHOS) and adenosine triphosphate (ATP) production catalyzing electron transport via reversible oxidation states of iron and providing a constant supply of energy necessary for normal heart function [1,59]. Data suggest that nearly one-third of cardiomyocyte iron is distributed in mitochondria, whereas cardiomyocyte mitochondria have 50–150% more iron compared to other cells [60]. Consequently, mitochondrial iron homeostasis must be tightly controlled as iron deficiency could severely disrupt mitochondrial energetics, whereas iron overload could result in mitochondria damage through ROS production. ROS react with iron in mitochondria and produce extremely deleterious hydroxyl radicals [19], followed by the depolarization of the mitochondrial membrane potential [61] and opening of the mitochondrial permeability transition pore, resulting in mitochondrial swelling [62].

The mitochondrion can use iron that has been imported into the cell using Tf-TfR complex or iron released from FT as a result of its degradation in proteasomes or lysosomes. The exact mechanism through which iron is transported across the outer mitochondrial membrane is under debate; however, evidence suggests that iron import into mitochondria is regulated via mitoferrin 2 (MFRN) and mitochondrial calcium uniporter (MCU) [30,62]. On the other hand, iron export is maintained via ABCB proteins such as ATP-binding cassette subfamily B member 8 (ABCB8) [63]. Cardiomyocytes also express a mitochondria-specific ferritin, FTMT, that has a high level of sequence identity with H-ferritin and is an important regulator of mitochondrial homeostasis since its loss results in mitochondrial oxidative damage [64].

Mitochondrial iron dysregulation has been linked with different cardiac pathological conditions, from cardiac ischemia (iron overload) to advanced heart failure (iron deficiency) [23,30,65]. These detrimental effects are partly mediated by the increased oxidative stress.

As iron is an important component of complexes of the respiratory chain, iron deficiency impairs mitochondrial complexes I–III, leading to reduction of ATP production [4]. In iron-deficient cardiomyocytes, expression of glycolytic enzymes is increased with a concomitant decrease in expression of Krebs cycle enzymes. However, the shift to glycolysis observed under these conditions is not sufficient to compensate for the loss in ATP production, resulting in a net decrease in ATP concentration and impaired function of ATP-dependent ion transport pumps. Furthermore, dysfunctional mitochondria are less likely to undergo mitophagy, accentuating the detrimental effect [4,59]. Enzymes that scavenge ROS also require iron for their production, and iron-depleted cardiomyocytes are more sensitive to ROS damage than cardiomyocytes with regular iron content [65].

Iron overload, either at the systemic or at the cellular levels, is also associated with harmful effects on cardiac function, as has been demonstrated in animal studies using mice with cardiomyocyte-specific deletion of the ferroportin gene as well as in humans with hemochromatosis or β-thalassemia [30,31]. Furthermore, patients with Freidreich ataxia, a disease characterized by mitochondrial iron overload in the heart and brain, develop dilated cardiomyopathy and arrhythmias [32]. The relative contribution of circulating “free” iron vs. intracellular LIP to the detrimental effect of iron is unclear.

Iron accumulation induces oxidative stress acting as a catalyst in Haber–Weiss and Fenton reactions, generating hydroxide ions (OH^−^) and hydroxyl radicals (HO^.^), which are very reactive and toxic free radicals [20]. These free radical by-products can also engage in secondary oxidation reactions in the cytosol and/or in the mitochondria and, in turn, damage DNA, proteins, and lipids. ROS can disturb cardiac intracellular Ca^2+^ homeostasis and affect multiple ion transporters responsible for myocardial electrical activity, leading to diastolic and systolic dysfunction and arrhythmogenesis [52]. Increased mitochondrial ROS generation results in depolarization of the mitochondrial membrane potential, opening of the mitochondrial permeability transition pore, leading to cell rupture and eventually to cardiac dysfunction and cardiomyopathy [66]. Although the exact mechanisms of cardiac mitochondrial injury induced by iron overload have not yet been fully elucidated, data suggest that treatment with the iron chelator, deferiprone, and the T-type calcium channel blocker, efonidipine, improves cardiac mitochondrial function [67].

## 4. Iron and Myocardial I/R Injury

Increased iron has been implicated in the pathology of I/R injury in a variety of organs including the heart. It has been postulated that during and following sustained ischemia, FT degradation prevails over FT synthesis with a subsequent release of iron [68]. Early studies have shown that relatively high concentrations of iron are mobilized into the coronary flow after prolonged ischemia, and this mobilization and redistribution of myocardial iron caused by ischemia may contribute to the oxidative damage and loss of cardiac function associated with the “reperfusion injury” [69]. Moreover, it has been demonstrated that even mild, non-overloading doses of iron (0.3–12 mg/mL, i.p.) can be detrimental to the heart under conditions of global I/R [70]. The highest dose of applied iron (12 mg/mL) caused a 17% reduction in post-ischemic recovery of left ventricular developed pressure, more than 50% decrease in cardiac work and cardiac output, associated with a two-fold increase in lipid hydroperoxides in the effluent. In addition to functional depression, iron-treated hearts also displayed myocardial injury manifested by two-fold higher post-I/R release of lactate dehydrogenase (LDH). During ischemia and early reperfusion, the acidotic and highly reduced intracellular environment favors release of ferric or ferrous iron from metalloproteins and facilitates iron-mediated Fenton chemistry with conversion of the less potent oxidants, superoxide and hydrogen peroxide, to the highly reactive strong oxidant, hydroxyl radical. In this context, administration of iron chelators, such as deferoxamine, beginning at the time of reperfusion, reduces the burst of oxygen free radical generation during the early minutes of reperfusion and the severity of reperfusion injury [71,72].

Increased TfR1 expression, due to activation of HIF signaling and subsequent increase of Tf uptake and iron accumulation, has been implicated as the mechanism responsible for the changes in cellular iron [73]. Furthermore, excessive free iron and the resultant oxidative stress and myocardial cell death observed in mice after MI was attributed to the downregulation of FTH protein, resulting in a reduced ability of cardiomyocytes to bind free intracellular iron [74]. More recently, increased mitochondrial iron was observed after cardiac I/R injury in mice and in cardiac tissue samples from patients with ischemic cardiomyopathy [23]. In addition, in the same study, genetic modification through overexpression of mitochondrial iron export protein ABCB8 was shown to be effective in a mouse model, due to an improved iron export, leading to protection against I/R injury as manifested by lower expression of cardiac stress markers and reduced cellular damage [23].

Depletion of iron by using iron chelators has been used as a cardioprotective approach to suppress ROS production. However, the benefits of using iron chelation in I/R injury are still debated. In some animal models, iron chelation therapy improves contractile function, increases cell viability, attenuates cardiac remodeling, and also reduces the size of infarction after I/R injury [75,76,77]. However, these results were not reproduced in larger animals [78,79,80]. A potential reason for the discrepant results may be tissue penetrance of the chelator used. Deferoxamine (DFO), which was used in the aforementioned studies, predominantly exerts its effect through iron binding in the extracellular space and endosome [81], whereas it cannot modulate mitochondrial iron [23].

Based on the preclinical studies, in which chelation of iron decreased the extent of I/R injury and/or myocardial infarct size in animal models, modulation of iron levels was investigated as a potential background for clinical treatment of I/R injury and reduction of myocardial infarct size (IS) in patients. Chan et al. (2012) [82] investigated whether DFO administered before reperfusion by primary percutaneous coronary intervention ameliorates oxidative stress and myocardial IS. The results of that trial have shown that adjunctive DFO treatment after the onset of ischemia and continued periprocedurally ameliorated oxidative stress without limiting IS. In support of this, pharmacological modulation of mitochondrial iron with the mitochondrial-permeable iron chelator, 2,2′-bipyridyl, protected against myocardial I/R injury, which may provide a novel therapeutic target against ischemic heart disease [23].

Several clinical studies have been performed to show the benefits of chelation therapy in patients with coronary artery disease (CAD). Thus, in the study by Paraskevaidis et al. (2005) [83], DFO was administered during coronary artery bypass grafting surgery, and this intervention protected myocardium against reperfusion injury, reduced patients’ stays in intensive care units, and also decreased lipid peroxidation [83]. In addition, positive effect of DFO treatment was manifested by and improved endothelium-dependent vasodilation in patients with CAD [84]. Furthermore, other chelators have been studied with the aim to improve post-MI myocardial function. In accordance, chelation therapy using EDTA (ethylene diamine tetraacetic acid) has been reported to attenuate adverse cardiovascular outcomes in patients with acute MI [85].

Taken together, mitochondrial iron is an important contributor in cardiac ischemic damage and may be a new therapeutic target in the management of ischemic heart disease [58]. Further studies are required in order to establish therapies that target iron more efficiently in cardiovascular diseases, especially in patients with acute MI.

## 5. Heme Oxygenase System in I/R Injury and Cytoprotection

### 5.1. Function of Heme Oxygenase

Heme oxygenase (HO) is an enzyme catalyzing the conversion of heme to CO, free iron, and biliverdin, while this reaction is the rate-limiting step in heme degradation. Three HO isoforms have been identified so far: HO-1, also known as heat shock protein 32, the inducible isoform present in the whole body but mainly in the spleen, liver, and kidneys; HO-2, an isoform constitutively expressed under physiological conditions, mainly in the testes, endothelial cells, and the brain; and HO-3, also constitutive but less characterized isoform of the enzyme [86,87]. Since heme conversion catalyzed by HO produces Fe^2+^, HO substantially contributes to iron metabolism in the body including the cardiovascular system. Among HO isoforms, HO-1, with relatively low expression in most tissues under physiological conditions, seems to be the most important isoform in the diseased states. HO-1 is significantly upregulated under stress conditions and contributes to the cytoprotective mechanisms via maintaining redox homeostasis during different forms of cellular stress such as ischemia, hypoxia, inflammation, or radiation [87,88,89]. Increased expression of HO-1 is mediated by the redox-sensitive transcription factor, Nrf2, through binding to and activating expression of antioxidant response elements (ARE) on the promoter region [90].

Breakdown products of HO-1, CO, and biliverdin, which were once considered to be toxic metabolic waste products, have been also shown to participate in cytoprotective signaling [87,91]. Maintaining low iron concentrations via increased FT levels plays an important role in cellular antioxidant defense and cytoprotection under stress conditions [87]. In line with this, overexpression of FTH was found protective via its anti-apoptotic effects in hepatic I/R injury [92].

### 5.2. HO-1 in Cardioprotection against Myocardial I/R Injury

Activation of HO-1 has been extensively documented to play a protective role in cardiac I/R injury achieved by interventions targeting oxidative stress in different experimental models of myocardial I/R injury. For example, activation of HO-1 is involved in post-I/R infarct size limitation and amelioration of left ventricle (LV) dysfunction afforded by several natural antioxidants such as polyphenols, the most studied of which is resveratrol. The cardioprotective effects of resveratrol against I/R injury were suggested to be mediated through HO-1/VEGF (vascular endothelial growth factor) pathway in rat nonpathological myocardium [93] and in diabetic [94] and hypercholesterolemic myocardium [95] and were associated with activation of phosphorylated protein kinase B (p-Akt), p-endothelial nitric oxide synthase (eNOS), and Mn superoxide dismutase (SOD). Resveratrol-mediated cardioprotective activation of Nrf2/ARE/HO-1 was also shown to be associated with enhanced activity of SOD and glutathione peroxidase (GPX) [96], stimulation of SIRT1, or inhibition of GSK3β [97]. Very recently, resveratrol was documented to protect myocardium against chronic, intermittent, hypoxia-induced injury via activating Nrf2/HO-1 signaling and blocking NLRP3 (the nod-like receptor family pyrin domain containing 3) inflammasome activation [98].

A variety of other substances have been shown to exert their cardioprotective effects against myocardial I/R injury via HO-1 activation. L-carnitine, either injected intraperitoneally to rats undergoing left anterior descending (LAD) coronary artery occlusion or administered to H9c2 cardiomyoblasts exposed to hypoxia/reoxygenation, reduced oxidative stress and apoptosis likely via activation of Nrf2/HO-1 pathway [99]. Galanthamine, an alkaloid with anti-inflammatory properties, has been shown to reduce myocardial I/R injury, endoplasmic reticulum stress-related apoptosis, and myocardial fibrosis in rats via activating 5′ adenosine monophosphate-activated protein kinase (AMPK)/Nrf2/HO-1 pathway [100]. Furthermore, atorvastatin, one of the most frequently used lipid-lowering drugs, statins, was shown to attenuate I/R-induced oxidative stress and inflammation in rat hearts via the Nrf2/HO-1 pathway, resulting in limitation of infarct size [101]. Recent studies documented that activation of HO-1 is associated with cardioprotection afforded by α_2_-adrenoceptor agonist dexmedetomidine [102], as well as by a natural compound, crocetin [103], or hydrogen-rich water [104]. Finally, an important role of HO-1 in cardioprotection against I/R injury was confirmed in HO-1 knock-out mouse hearts subjected to ex vivo I/R. In these hearts, the post-I/R infarct size and incidence of ventricular fibrillation were significantly increased when compared with WT mice [105].

Of the three main heme metabolites produced by HO-1, CO has been documented to exert cardioprotective effects in I/R. Pretreatment of donor rats by exposure to CO inhalation prevented I/R injury following heart transplantation [106] and suppressed apoptosis [107]. Similarly, CO released from CO-releasing molecules (CORM) attenuated I/R-induced apoptosis in cardiomyocytes via a mitochondrial pathway [108] and induced preconditioning-like cardioprotective and anti-apoptotic effects in mouse hearts subjected to in vivo I/R injury, mediated via nuclear factor NF-κB, signal transducer and activator of transcription (STAT1/3), and Nrf2 signaling [109].

**Figure 2:**
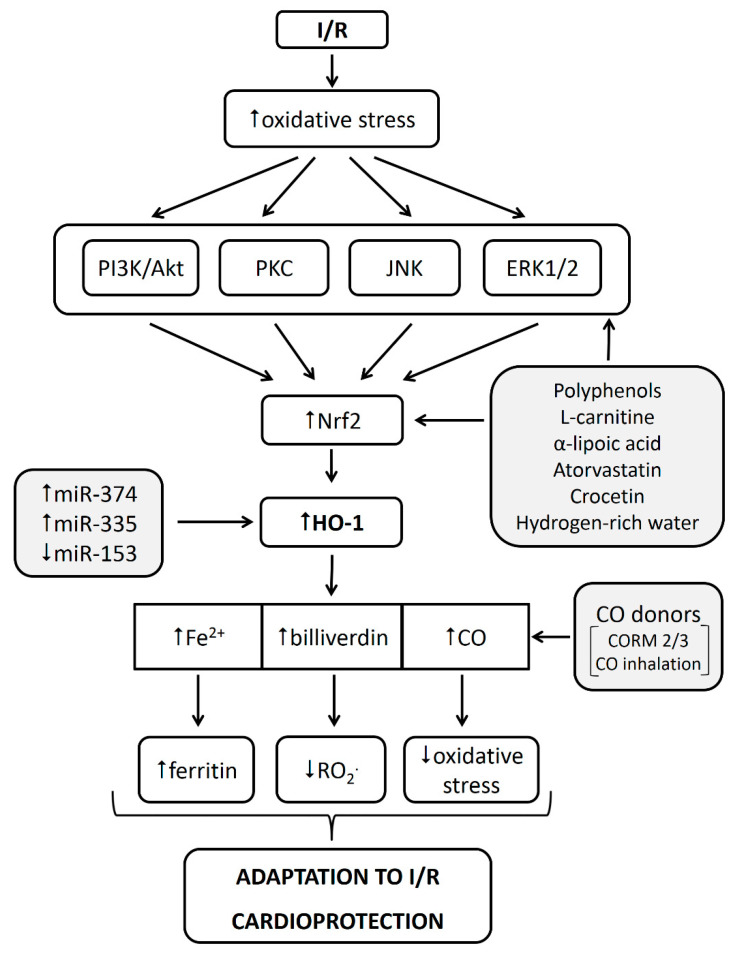
Role of HO-1 in I/R injury and cardioprotection. Cardiac I/R induces oxidative stress that consequently activates intracellular adaptive mechanisms in cardiomyocytes such as PI3K/Akt, PKC, JNK, or ERK1/2 signaling pathways. They further activate Nrf2 as the major positive regulator of HO-1 in the cell. Nrf2/HO-1 pathway can be activated also by application of several cardioprotective substances including polyphenols, L-carnitine, atorvastatin, crocetin, or hydrogen-rich water. HO-1 products (Fe^2+^, biliverdin, CO) then can scavenge free radicals, thus attenuating oxidative stress, which, in result, leads to cardioprotection. Finally, CO can be increased by direct application of CO donors, which, in turn, also leads to cardioprotection. Abbreviations: I/R, ischemia/reperfusion; PI3K, phosphatidylinositol-3-kinase; JNK, c-Jun NH2-terminal protein kinase; ERK1/2, extracellular signal regulated kinase 1/2; Nrf2, nuclear factor erytroid 2-related factor 2; HO-1, heme oxygenase-1; miR, microRNA; CO, carbon monoxide; CORM, CO-releasing molecules; RO_2_^.^, peroxyl radical.

Taken together, HO, as the key enzyme involved in iron metabolism in the organism, has been shown to be intimately involved in adaptation of the heart to different pathological stimuli including myocardial I/R. Particularly, the inducible HO-1 isoform is upregulated in conditions of myocardial ischemia as well as after stimulation by diverse cardioprotective interventions such as administration of polyphenols, L-carnitine and others, mainly via activation of phosphatidylinositol-3-kinase (PI3K)/Akt, eNOS, protein kinase C (PKC), Nrf2/ARE pathway. HO-1 activation leads to increased production of three main heme metabolites, Fe^2+^, biliverdin, and CO, among which particularly CO has been documented to exert cardioprotective effects in I/R. HO-1 activation is associated with decreased oxidative stress and anti-apoptotic and anti-inflammatory effects, finally leading to enhanced ability of the myocardium to resist against I/R injury (Figure 2).

Thus, HO-1 activation may represent a promising therapeutic strategy to prevent myocardial I/R injury in patients suffering from ischemic heart disease or myocardial infarction as well as to enable better preservation of hearts during transplantation.

## 6. Role of Iron and Ferritin in Heart Preconditioning

In the last decades, research has demonstrated a robust efficiency of ischemic preconditioning (IPC) as a short-term adaptive phenomenon protecting myocardium and/or other organs of all animal species including humans against prolonged ischemia by way of adaptation to brief episodes of ischemia [110,111]. However, molecular mechanisms of IPC are very complex and not yet fully elucidated. It is well known that keeping of iron in FT prevents iron-dependent formation of free radicals [112]. Therefore, several studies have been designed to explore whether iron and FT could play a role in cardioprotection afforded by different forms of conditioning.

As mentioned above, even moderate concentrations of iron could cause post-I/R deterioration of heart function and increased ROS production [70]. On the other hand, preconditioning protected severely stressed hearts (40 min ischemia/15 min reperfusion) by modulating tissue iron status and distribution, as well as iron-catalyzed production of radicals. Further studies unraveled the role of iron in the IPC mechanisms, and it has been proposed that iron may play a dual role in myocardial injury [68]. While high levels of iron mobilized following sustained ischemia were detrimental, increasing susceptibility of cardiac tissue to oxidative damage, iron also served as a signaling molecule for the accumulation of FT. IPC produces small, nontoxic but stimulating amounts of “free” iron, which enhance FT formation (through conversion of the iron-responsive proteins IRP-1 to the CA and do not promote its degradation. This iron is not involved in cardiac injury, but rather prepares the heart for the forthcoming action of high levels of “free” iron after prolonged ischemia. The concept of “Fe preconditioning” was introduced by Galleano et al. (2011) [113] who demonstrated that subchronic, low-level iron administration protected the liver against I/R injury (also by upregulation of FT content) that was associated with the recovery of the NF-κB signaling which was lost during I/R.

Low levels of “free” iron generated in the cell during and following IPC stimulus initiated FT translation resulting in its de novo synthesis [114]. FT accumulation reached 359% of its basal level and it remained high during the subsequent period of prolonged ischemia, when an increase in L-ferritin mRNA was observed, indicating that the transcriptional mechanism of FT synthesis was activated. The increased amounts of intracellular FT sequester excessive catalytic iron and prevent oxidative damage. During the reperfusion phase, the newly synthesized FT binds (scavenges) the labile iron released during ischemia and thus protects the heart against the deleterious iron-catalyzed free radicals. At this stage, the amount of FT decreased to 178% of its basal level and the ratio of its L and H subunits also returned to the pre-ischemic value. The increase in FT content evoked by IPC and the essentiality of an iron signal in the IPC-induced protective mechanism has been thus suggested as a potential mechanism of cardiac protection. It was also confirmed by the discovery that selective iron chelators (acetyl hydroxamate or Zn-desferrioxamine) abrogated the functional protection and suppressed FT accumulation [114].

Identification of the source of the “iron signal” suggested that it could stem from at least three sources: (1) Heme catabolism by heme oxygenases, (2) degradation of ISCs, and (3) degradation of iron-containing proteins, mainly FT [115]. Degradation of FT could potentially serve as the main source for the “iron signal” because breakdown of a single FT molecule could release more than 1200 iron ions into the cytoplasm. Degradation of intracellular proteins is mediated by either lysosomal proteases and/or the proteasome pathway. This hypothesis was confirmed by adding the proteasome inhibitor MG132 to KH-buffer prior to the IPC procedure, which resulted in the loss of the IPC-induced protection against I/R injury and in the inhibition of the cytosolic FT degradation and release of iron [115]. MG132 can inhibit proteasome-mediated IRP degradation and, as such, reduces the translation of FT mRNA. It was also found that during a 30-min “delay” after IPC prior to prolonged ischemia, FT broke down by lysosomal proteases, resulting in a decrease in cardiac hemodynamic recovery. In addition, when I/R was separated from the IPC by this “delay”, FT mRNA levels remained stable during the whole experiment including ischemia. Thus, apparently the time gap between the IPC and the I/R may be crucial for the regulation and expression of FT.

**Figure 3:**
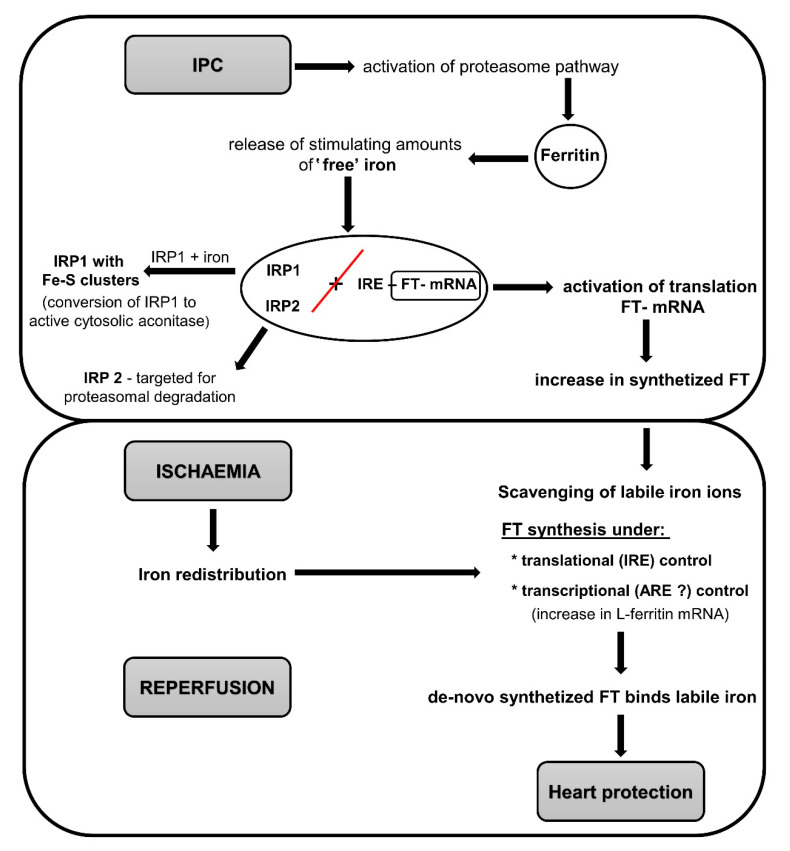
Schematic representation of the mechanisms of cardioprotection conferred by IPC based on iron and ferritin production. In the first step, IPC activates proteasome pathways, which degrade molecules of FT and release small and nontoxic amounts of “free” iron. This iron serves as a signaling molecule for the accumulation of FT. The expression of FT is post-transcriptionally regulated by iron-regulatory proteins (IRPs), IRP1 and IRP2. When intracellular iron is low, IRP1 and IRP2 bind with high affinity to the iron-responsive element (IRE) within the ferritin mRNA and thus inhibiting its translation. Under IPC conditions when iron is abundant, IRP1 combines with this free iron and dissociates IRP1 and 2 from the IRE (shown by red line), thus allowing ferritin mRNA translation (de novo translation) and accumulation of FT. IRP1 is converted into active cytosolic aconitase, and IRP2 is targeted for proteasomal degradation. Under normal conditions, during and following sustained ischemia, FT degradation prevails over FT synthesis, leading to the release of iron, subsequently increasing the susceptibility of cardiac tissue to oxidative damage. On the other hand, after the application of IPC, FT levels remained high during the subsequent period of prolonged ischemia, and an increase in L-ferritin mRNA was observed, indicating that the transcriptional mechanism of FT synthesis was activated. During the reperfusion phase, the newly synthesized FT scavenges the labile iron released during ischemia and thus protects the heart. Abbreviations: ARE, antioxidant response element, Fe–S clusters, iron–sulfur clusters; FT, ferritin; IPC, ischemic preconditioning, IRE, iron responsive element; IRP1, iron regulatory protein 1; IRP2, iron regulatory protein 2; Red line indicates dissociation of IRP1 and 2 from the IRE.

The obtained cumulative information concerning the involvement of iron and FT in the mechanisms of IPC and cardioprotection is summarized in Figure 3.

### 6.1. Role of Iron and Ferritin in Other Cardioprotective Phenomena

The important role of iron and FT in protection against I/R injury has been demonstrated in other forms of conditioning and in organs other than the heart. Thus, hypothermic preconditioning of human coronary artery endothelial cells (HCAECs) by incubation at 25 °C induced an increase in cell iron and a significant rise in FT levels. This was associated with protection of HCAECs against cold-induced, iron-mediated oxidative stress and attenuated cell injury caused by storage at 0 °C [116]. Similarly, protection against lethal I/R injury induced by intermittent hypobaric hypoxia (8 h/day for 4 weeks) in rats was associated with a decrease of iron in tissues (heart, liver, spleen, kidney) and in plasma, as well as with increased erythropoiesis and the downregulation of hepcidin expression [117].

Chronic metabolic preconditioning has been proposed as one of the reasons of enhanced resistance to I/R injury in the diabetic hearts and a failure to further precondition them with IPC [118,119,120,121,122]. This hypothesis has been confirmed in the study by Vinokur et al. (2013) [123], which suggested that the lost efficiency of IPC in the diabetic heart might be related to the modulation of iron homeostasis. Indeed, basal FT levels, which are two-fold higher in the diabetic heart compared to nondiabetic controls, decrease rapidly and dramatically (four-fold) during prolonged ischemia and reperfusion (with prior IPC) in the diabetics but not in the controls. This study, thus, indicated why subjecting the diabetic heart to IPC need not confer protection against I/R injury. Similar to that, anti-infarct protection by postconditioning with sevoflurane (sevo-postC) was not effective in the diabetic hearts, in contrast to its effect in nondiabetic hearts [124]. However, sevo-postC-mediated cardioprotection in controls did not involve the de novo FT synthesis and accumulation.

The recent study by Mieszkowski et al. (2019) [125] was the first study on human subjects aimed to investigate the effects of remote ischemic preconditioning (RIPC) on peripheral blood mononuclear cells (PBMC). They investigated the effect of acute (1-day) and 10-day RIPC (upper limb RIPC: four cycles, 5 min ischemia/5 min reperfusion) on the Wingate Anaerobic Test ((WAnT) measures relative peak power and relative mean power (W/kg)) on the FTH, FTL, and TfR1 mRNA expression in PBMC, and anaerobic performance. Ten days OF RIPC, unlike one day of RIPC, significantly increased upper limbs’ relative mean power and also caused significant increase of FTH and FTL mRNA and decrease in TfR1 mRNA. On the other hand, acute RIPC resulted in a significant decrease in FTH, FTL, and TfR1 mRNA levels.

### 6.2. Ferritin and Protection by NO Donors

It has been previously shown that part of the molecular mechanisms of IPC is associated with nitric oxide (NO) and its synthases, such as eNOS and inducible NOS (iNOS). The study by Grievink et al. (2016) [126] demonstrated that NO generated by exogenous NO-donors sodium nitroprusside (SNP), S-nitroso-N-acetyl-dl-penicillamine (SNAP), and 3-morpholinosydnonimine (SIN-1), as a form of pharmacological preconditioning) could play a role in “iron-based” mechanism of cardioprotection. Pretreatment with 10 μM SNAP significantly reduced IS and increased CA activity and FT accumulation, while pretreatment with 2 μM SIN-1 increased IS and was associated with lower FT protein levels. Pretreatment with SNP did not cause any changes. These findings indicate that exogenous NO (similar to IPC), depending on its concentration and bio-active redox form, can regulate iron metabolism (through FT accumulation) and may be involved in the “iron-based” mechanism of cardioprotection.

In conclusion, ischemic preconditioning initiates the de novo synthesis of FT in the heart; the extra FT is proposed to serve as a ‘sink’ for redox-active iron, thus protecting the heart from iron-mediated oxidative damage associated with I/R injury. These data substantiate a novel iron-based mechanism of ischemic preconditioning and could pave the way for the development of new modalities of heart protection.

## 7. Iron and Ferroptosis: A Less-Known Form of Cell Death and Its Mechanisms

Ferroptosis, known as simultaneously occurring and mutually amplifying accumulation of redox-active iron, glutathione depletion, and lipid peroxidation [127], was identified in 2012 [128] and since that time it has been documented in several cardiac pathologies, including doxorubicin-induced cardiomyopathy [77] and acute I/R injury under conditions without metabolic disorders [77,129] as well as with diabetes [130,131], post-myocardial infarction heart failure in the early and middle stages [132], and in septic heart injury [133]. At a cellular level, ferroptosis has been found in various models of cardiomyocytes, such as H9c2 cells subjected to hypoxia/reoxygenation with normal and high glucose levels [130,131] and rat ventricular myocytes [132].

Ferroptosis is morphologically, biochemically, and genetically distinct from other modes of regulated cell death. It can be induced by experimental small molecules (e.g., erastin, Ras-selective lethal small molecule 3, and sulfoximine), and some drugs (e.g., sulfasalazine, sorafenib, and artesunate) [134,135]. The most frequently described pathway of ferroptosis induction by erastin involves the inhibition of cystine uptake by the cystine/glutamate antiporter, leading to suppressed antioxidant defenses due to glutathione reduction [128,136]. Glutathione, being converted from a reduced form (GSH) to the oxidized one (GSSG), is used by the phospholipid peroxidase glutathione peroxidase 4 (GPX4) to convert polyunsaturated fatty acids’ hydroperoxides formed in membrane phospholipids by lipoxygenase (LOX) enzymes to their corresponding, less harmful, lipid alcohols [137]. The direct inhibition or indirect inactivation of GPX4 by various endogenous molecules (e.g., selenium, dopamine, vitamin E, CoQ10,) and chemicals (e.g., ferrostatin-1, dexrazoxane) [134,135] results in oxidative stress. In the case of the accumulation of iron, which is required for the normal activity of LOX enzymes [138], this GPX4 inhibition promotes overwhelming lipid peroxidation, terminating in lethal ferroptosis cell damage. Phosphatidylethanolamines have been identified to be key phospholipids driving ferroptosis due to peroxidation [139]. The location in which lipid peroxidation takes place during ferroptosis remains an unresolved question. However, it has been suggested that the mitochondria [77,140], endoplasmic reticulum [139], and lysosomes [141] are mainly affected, while the involvement of plasma membrane is controversial in this respect [77,142]. In addition to the main aforementioned regulatory mechanisms, other molecules can also affect lipid peroxidation and potentially ferroptosis execution. In fact, selenium is required for the biosynthesis of GPX4 [143] and NADPH (Nicotinamide adenine dinucleotide phosphate) acts as a reductant needed to eliminate lipid hydroperoxides and, thus, it is considered as a biomarker of ferroptosis sensitivity [144]. Likewise, since coenzyme Q_10_ has been shown to be depleted by a ferroptosis inducer [145], this membrane antioxidant can also modulate cellular sensitivity to this cell death.

### 7.1. Ferroptosis and Post-I/R Myocardial Injury

Although early reperfusion affords protection to ischemic myocardium, in patients with acute MI with ST-segment-elevation (STEMI) undergoing percutaneous coronary intervention (PCI), myocardial hemorrhage is a common complication after reperfusion intervention, limiting its success [146,147]. It is associated with residual myocardial iron in post-infarcted area [148] and is capable to potentiate increased ROS production and ferroptosis [149]. Using cardiac magnetic resonance (CMR) imaging, Robbers et al. (2013) [150] indicated that hemorrhage representing an irreversible microvascular destruction might be preceded by microvascular obstruction, and this relation was further confirmed by Carrick et al. (2016) [147] in STEMI patients. Moreover, associations between myocardial hemorrhage revealed by CMR and the severity of MI in the acute phase, as well as with the development of adverse left ventricle (LV) remodeling in the long term (defined as an increase in LV end-diastolic volume and reduced LV ejection fraction), were found These studies [146,147,148] clearly demonstrated the deleterious role of residual myocardial iron in some post-infarcted patients after primary PCI on the one hand, and, on the other hand, pointed to a potential of cardiomyocytes‘ salvage through the targeting ferroptosis and regulation of ROS production using protective effects of the mechanistic target of rapamycin (mTOR) [149].

### 7.2. Ferroptosis as a Potential Novel Target for Cardioprotection

As mentioned above, ferroptosis has been found to underlie lethal injury of the heart due to both acute and chronic I/R and in various types of cardiomyopathies [77,102,129,130,131,132]. In contrast, targeting ferroptosis has been suggested as a feasible approach for managing these cardiac pathologies. Genetic manipulations in ferroptosis signaling, such as overexpression of Slc7a11, a key component of the cystine-glutamate antiporter [151], knockdown of *Nrf2*, which regulates HO-1 expression [77] and depletion of GPX4 [132], have been shown to increase resistance against ferroptosis and alleviate damage of the heart and cardiac myocytes. Consistently with these results, pharmacological interventions, such as iron chelation, have also shown effective protection against iron-dependent processes of cell death. It has been demonstrated that iron chelator dexrazoxane (DZX), as a mitochondria-permeable metal chelator, decreased free radicals’ generation and improved post-I/R hemodynamics in the ex vivo rat hearts [152]. In addition, DZX protected mice against DOX-induced ferroptosis and reduced lethal heart injury (size of infarction) and myocardial dysfunction following I/R [77]. Among commercially available chelators, deferoxamine (DFO) is the most widely used nontoxic iron chelators to treat patients with different diseases associated with iron overload. Furthermore, DFO has been shown to reduce ROS generation in rat cardiomyocytes [153]. Protective effects have been also demonstrated in other excess iron-induced cell death and ferroptosis models [149] using ferroptosis inhibitor ferrostatin-1 acting through scavenging of alkoxyl radicals produced by ferrous iron from lipid hydroperoxides [154]. In line, ferrostatin-1 exhibited beneficial effects in mice with cardiomyopathy due to FTH-deficiency [151].

Other interventions with anti-ferroptotic effects include activation of mTOR [149] and inhibition of HO-1, causing heme degradation with resultant release of free iron (inhibitor zinc protoporphyrin IX) [77,151,154], as well as other lipid peroxidation inhibitors (liproxstatin-1) [129]. In summary, these findings concerning ferroptosis confirm a paradigm of an important role of nonapoptotic cell death in the heart [130,155,156,157] and indicate that pharmacological interventions targeting one of these necrotic-like cell death modes or multi-target approaches can affect cardiovascular mortality at a greater extent than it is in the case of caspase modulation. This is also supported by the evidence showing that ferroptosis can accompany necroptosis and pyroptosis in the heart [130,157].

## 8. Conclusions

Iron is an essential mineral that plays pivotal roles in both normal physiological processes and in pathological mechanisms underlying a variety of diseases. The issue concerning the relationship between iron and cardiovascular disease has been discussed for the last four decades, starting with a study of Sullivan published in 1981, and is still not definitely resolved. Although studies constantly bring novel findings concerning the mechanisms of iron-related deleterious effects on the heart linked with ROS production and increased susceptibility to myocardial injury, especially under conditions of ischemia and reperfusion, the association of iron, as a potential risk factor, and cardiovascular diseases remains elusive.

Besides being a catalyst for cell death mechanisms through the enhanced ROS production including ferroptosis, iron also acts as a signal triggering a variety of protective cellular cascades. In particular, the role of iron in the mitochondria is especially important, and its management may contribute to a more effective treatment of myocardial I/R injury. Further studies are needed to elucidate mechanisms of cellular iron homeostasis that may lead to development of novel and more efficient iron-targeting therapies of cardiovascular diseases, in addition to iron chelation.

## Abbreviations

ABCB8	ATP-binding cassette subfamily B member 8 protein
ARE	Antioxidant response elements
BMP-SMAD	Bone morphogenetic protein/small mothers against decapentaplegic pathway
CA	Cytosolic aconitase
CO	Carbon monoxide
DFO	Deferoxamine
DMT-1	Divalent metal transporter 1
Fe^2+^, Fe^3+^	Ferrous iron, ferric iron
FT	Ferritin
FTH	Ferritin heavy chain
FTL	Ferritin light chain
FPN	Ferroportin
FTMT	Mitochondria-specific ferritin
GPX	Glutathione peroxidase
GPX4	Phospholipid peroxidase glutathione peroxidase 4
GSH	Reduced form of glutathione
GSSG	Oxidized form of glutathione
HCAECs	Hypothermic preconditioning of human coronary artery endothelial cells
HIF	Hypoxia-inducible factor
HO^.^	Hydroxyl radicals
HO-1	Heme oxygenase-1
IPC	Ischemic preconditioning
I/R	Ischemia/reperfusion
IRE	Iron-Responsive elements
IRP1, IRP2	Iron regulatory proteins 1 and 2
IS	Infarct size
ISCs	Iron sulfur clusters
LIP	Labile iron pool
LOX	Lipoxygenase
MCU	Mitochondrial calcium uniporter
MFRN	Mitoferrin
NO	Nitric oxide
Nrf2	Nuclear factor erythroid 2-related factor 2
NTBI	Nontransferrin-bound iron
OH^−^	Hydroxide ions
PBMC	Peripheral blood mononuclear cells
RIPC	Remote ischemic preconditioning
RO	Alkoxyl radicals
RO_2_	Peroxyl radicals
ROS	Reactive oxygen species
Sevo-postC	Postconditioning with sevoflurane
SOD	Superoxide dismutase
Tf	Transferrin
TfR	Tf receptors
